# Case Report: Complete response to the novel NaPi2b-targeting ADC YL205 in heavily pretreated platinum-resistant ovarian cancer

**DOI:** 10.3389/fonc.2026.1781961

**Published:** 2026-05-21

**Authors:** Yan-Xiang Tang, Hui Xiao, Zhen-Zi Tang, Lian Jian, Xingzi Guo

**Affiliations:** 1 Department of Gynecologic Oncology, Hunan Cancer Hospital, The Affiliated Cancer Hospital of Xiangya School of Medicine, Central South University, Changsha, Hunan, China; 2 Department of Radiology, Hunan Cancer Hospital, The Affiliated Cancer Hospital of Xiangya School of Medicine, Central South University, Changsha, Hunan, China

**Keywords:** adverse events, antibody-drug conjugate, complete response, high-grade serous ovarian carcinoma, NaPi2b, platinum-resistant ovarian cancer, YL205

## Abstract

**Objectives:**

High-grade serous ovarian carcinoma (HGSOC) remains a formidable challenge in the platinum-resistant setting (PROC). In the post-PARP inhibitor (PARPi) era, prior exposure reshapes resistance mechanisms, necessitating a shift from traditional platinum-free interval definitions to biomarker-driven strategies. We report an exceptional response to a next-generation ADC and review the evolving therapeutic landscape.

**Case report:**

A 40-year-old woman with FIGO stage IVB, BRCA-wildtype HGSOC developed rapid multi-drug resistance following neoadjuvant chemotherapy, optimal cytoreduction, and progression on maintenance olaparib/bevacizumab, gemcitabine, and a USP1 inhibitor. Following the identification of high NaPi2b expression (83% tumor proportion score), she initiated YL205, a novel NaPi2b-targeting antibody-drug conjugate (ADC) utilizing a topoisomerase I inhibitor payload, at 2.0 mg/kg every 3 weeks.

**Results:**

The patient achieved a sustained complete radiological response (CR) and CA-125 normalization 3.5U/ml after 24 weeks, remaining stable through 48 weeks treatment. This represents the first clinical instance of CR observed with this agent. Adverse events were limited to manageable grade 1–2 neutropenia symptoms.

**Conclusions:**

This case underscores the potential of next-generation ADCs featuring optimized linker-payload technologies. By utilizing topoisomerase I inhibitors, agents like YL205 bypass microtubule-stabilization resistance induced by prior taxane exposure, while “bystander effects” address intratumoral heterogeneity. Longitudinal, biomarker-matched strategies and proactive toxicity management are essential to achieving deep tissue clearance in heavily pretreated HGSOC.

## 1 Introduction

Ovarian cancer is the eighth most common cause of cancer death in women worldwide. In 2025, the United States is expected to see 20,890 new diagnoses and 12,730 deaths (1). Approximately 80% of patients are diagnosed at stage III–IV, with 5-year overall survival ranging from 10–40%. Despite initial remission rates of ~80% with platinum-based therapy, ~75% of advanced-stage patients relapse within 2 years (2). Platinum-resistant ovarian cancer (PROC, platinum-free interval <6 months) has historically been associated with dismal outcomes, with standard non-platinum chemotherapy yielding ORRs of only 10–15% and median progression-free survival (PFS) of ~3.5 months (3, 4).

In the era of precision medicine, the widespread integration of maintenance therapies—specifically poly (ADP-ribose) polymerase inhibitors (PARPi)—has added significant complexity to the clinical management and definition of platinum-resistant ovarian cancer (PROC). Recent comprehensive analyzes underscore the evolving resistance patterns in PROC, with PARPi exposure altering tumor biology and reducing response to subsequent therapies (5–7). Standard treatments in PROC yield low response rates and short progression-free survival (PFS), necessitating novel approaches such as antibody-drug conjugates (ADCs) that target tumor-specific antigens. This case report details a young patient with aggressive, multi-resistant HGSOC who achieved a complete response to YL205, a NaPi2b-targeting ADC. What is unique about this case is the first reported complete radiological response to YL205 in a heavily pretreated patient, highlighting its potential to overcome resistance mechanisms in the post-PARPi era. Accompanied by a review of the literature focused on ADC advancements, we discuss diagnostic and management challenges, the evolving therapeutic landscape of ADCs in PROC, including their efficacy and safety profiles, and the critical role of biomarker-driven strategies.

## 2 Case presentation

Patient Information: A 40-year-old woman presented in late April 2023 after self-palpating a firm, non-tender left cervical lymph node measuring approximately 1 cm.

### 2.1 Clinical findings and diagnostic workup (April–May 2023)

Initial evaluation at local hospitals revealed significantly elevated tumor markers: Cancer Antigen 125 (CA-125) at 8956.00 U/mL and Human Epididymis Protein 4 (HE4) at 263.04 pmol/L. Contrast-enhanced computed tomography (CT) scans identified a left adnexal mass, suggestive of an ovarian primary, alongside extensive metastatic lymphadenopathy involving the left cervical, and retroperitoneal regions. A subsequent ultrasound-guided biopsy of the left cervical lymph node was performed on May 5, 2023. Histopathological examination confirmed metastatic adenocarcinoma. Immunohistochemistry (IHC) profile [CK7+, WT-1+, Pax-8+, p53 nonsense mutation, ER focal+] was consistent with a high-grade serous ovarian carcinoma (HGSOC) origin. The patient was staged as FIGO IVB.

## 3 Treatment timeline outcomes

### 3.1 Initial therapy and surgery (May–August 2023)

The patient received three cycles of neoadjuvant chemotherapy with carboplatin and paclitaxel (TC regimen) from May to July 2023, achieving a partial radiological response and a reduction in CA-125 to 446 U/mL. She underwent optimal interval cytoreductive surgery on August 4, 2023, which included total hysterectomy, bilateral salpingo-oophorectomy, pelvic and para-aortic lymphadenectomy, omentectomy, and splenectomy. Intraoperative assessment confirmed an intra-abdominal optimal debulking surgery (R0). Post-operative pathology confirmed HGSOC with metastatic involvement in one para-aortic lymph node and the splenic hilum.

### 3.2 Post-operative course and early-line chemotherapy (August 2023 –February 2024)

The post-operative period was complicated by recurrent small bowel obstructions, necessitating conservative management and eventual surgical adhesiolysis on October 29, 2023. Adjuvant chemotherapy was resumed but modified due to biochemical progression (rising CA-125). She received sequential lines including paclitaxel/oxaliplatin/bevacizumab (October 2023). Due to a plateau in CA125 levels, the treatment was changed to weekly nab-paclitaxel with bevacizumab (December 2023 onwards). By February 19, 2024, CA-125 normalized, and imaging showed stable disease.

### 3.3 Maintenance therapy and progression (April–November 2024)

Given confirmed homologous recombination deficiency (HRD) positivity with BRCA wild-type status, maintenance therapy with olaparib (300mg twice daily) and bevacizumab was initiated in April 2024. However, biochemical progression (rising CA-125) was noted by July 2024. By November 2024, CT imaging confirmed disease progression with enlarging multiple metastatic lymph nodes in the left lower cervical and retroperitoneal.

### 3.4 Subsequent therapies and clinical trial enrollments (November 2024–March 2025)

• Gemcitabine Monotherapy (November 2024): She received two cycles of gemcitabine but experienced rapid biochemical progression (CA-125 rising from 157.9 to 326.9 U/mL within a month).

• Investigational USP1 Inhibitor (SIM0501) Trial (December 2024 - March 2025): The patient was enrolled in a phase I trial of SIM0501. After approximately three months of treatment, both serological (CA-125 rising to 954.59 U/mL) and radiological progression were confirmed, leading to trial discontinuation.

### 3.5 Biomarker profiling and YL205 therapy (April 2025–present)

Comprehensive biomarker profiling performed in March 2025 revealed high NaPi2b expression (83% tumor proportion score by IHC), with low human epidermal growth factor receptor 2 expression (HER2 1+). The NaPi2b IHC assay (TPS 83%) was conducted on archival FFPE sections originally biopsied from the left cervical lymph node in May 2023. To ensure protein stability, new 4μm sections were prepared on April 3, 2025, and verified by a central laboratory for tissue viability. This approach is supported by evidence indicating the temporal stability of NaPi2b expression in HGSOC. Based on this profile, the patient was enrolled in a phase I/II trial of YL205, a novel NaPi2b-targeting ADC with a topoisomerase I inhibitor payload. Treatment was initiated on April 14, 2025, at a dose of 2.0 mg/kg every three weeks.

**Figure 1:**
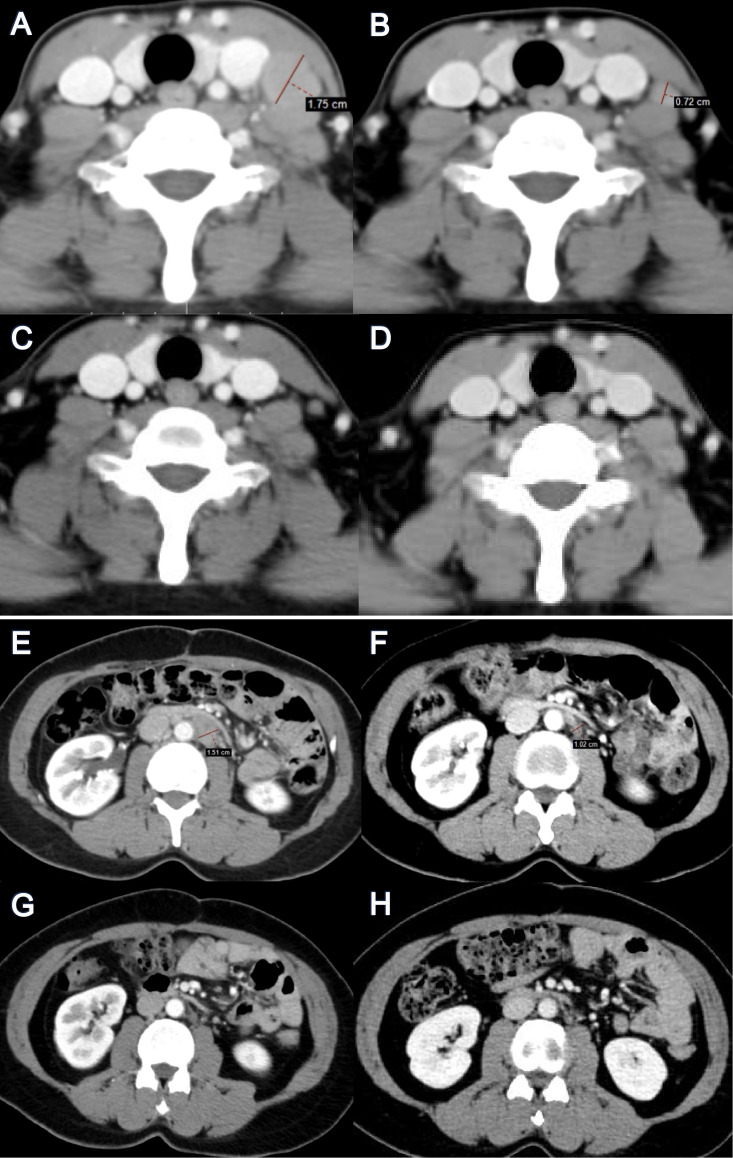
Serial CECT imaging demonstrating sustained complete response to YL205 in platinum-resistant ovarian cancer. Panels **(A–D)** show the left cervical lymph node metastasis, and panels **(E–H)** show the retroperitoneal lymph node metastasis, imaged at four key time points: **(A, E)** baseline (March 2025), demonstrating enlarged metastatic lymph nodes (1.75 cm and 1.51 cm in short-axis diameter, respectively); **(B, F)** Week 6 (June 2025), showing partial response with reduced lesion size (0.72 cm and 1.02 cm); **(C, G)** Week 24 (September 2025), confirming complete radiological response (CR) with full resolution of the lesions; and **(D, H)** April 2026 (latest follow-up), showing sustained CR with no evidence of disease recurrence. All assessments were performed using systemic CECT in accordance with RECIST 1.1 criteria.

**Figure 2:**
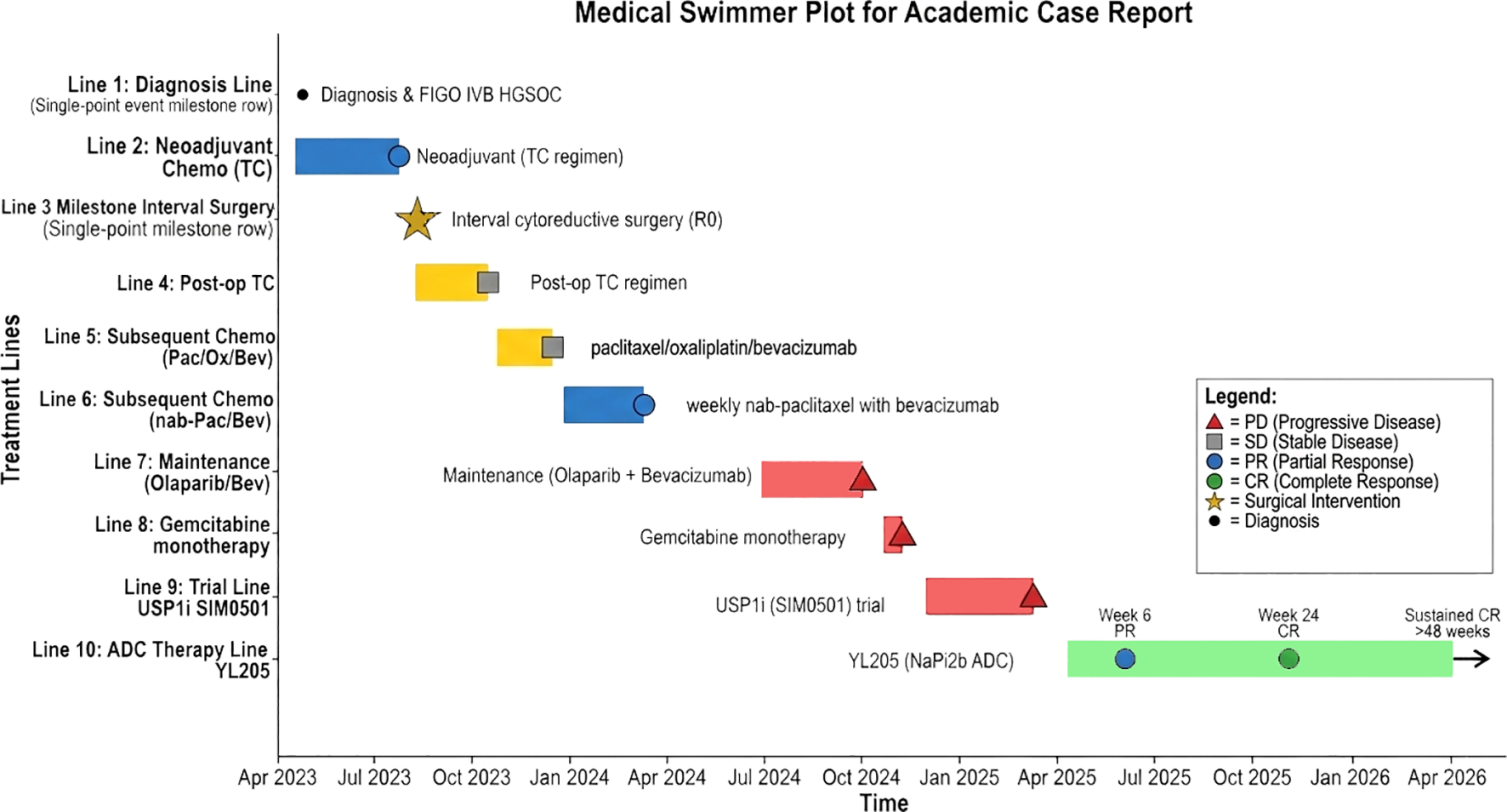
Swimmer plot of the patient’s treatment journey and clinical response. The plot spans from initial diagnosis (April 2023) to the latest follow-up (April 2026). Each row represents a distinct treatment line or key clinical event, with horizontal bars indicating treatment duration. Responses are color-coded: blue = partial response (PR), yellow = stable disease (SD), red = progressive disease (PD), green = complete response (CR). Symbols mark key events: black circle (diagnosis), gold star (R0 interval surgery), blue/green circles (PR/CR onset), red triangles (PD), and arrow (ongoing response). All prior therapies failed to achieve durable disease control, whereas the NaPi2b-targeted ADC YL205 induced PR at Week 6, confirmed CR at Week 24, and sustained CR for >48 weeks as of follow-up.

• Response: By the first tumor assessment after 6 weeks (June 2025), CA-125 had normalized to 16 U/mL, and systemic contrast-enhanced CT (covering all baseline metastatic sites) showed a partial response. After 24 weeks (September 2025), a complete radiological response (CR) was confirmed, with resolution of all previously measurable lymph node metastases (**Figure 1**), accompanied by a CA-125 level of 3.5 U/mL. This CR has been sustained through the most recent follow-up in April 2026. The patient’s full treatment journey, including all prior lines of therapy and corresponding clinical responses, is comprehensively visualized in the Swimmer plot (**Figure 2****).**

• Tolerability: The patient maintained the full dose of 2.0 mg/kg YL205 for 48 weeks without interruption. The primary hematological event was persistent but manageable Grade 2 leukopenia. Following an early nadir of 1.1*10^9/L (May 6, 2025), the leukocyte counts demonstrated a fluctuating recovery pattern, typically ranging between 1.2*10^9/L and 1.9*10^9/L throughout the maintenance phase. This hematological toxicity was limited to the white cell lineage, with no significant impact on hemoglobin or platelet levels. The absence of Grade 3 events or febrile neutropenia further underscores the safety of long-term YL205 administration.

### 3.6 Patient perspective

The patient reported initial anxiety upon diagnosis and during multiple treatment failures and complications like recurrent obstructions, but expressed profound relief and improved quality of life following the response to YL205. She noted, “After so many treatments that didn’t work and surgeries for obstructions, starting YL205 brought hope. The side effects were mild compared to chemotherapy, and seeing the tumors disappear on scans was life-changing. I’m grateful for the team’s support and access to this trial.”

## 4 Discussion

### 4.1 Redefining platinum resistance

This patient’s disease progression during PARPi maintenance therapy exemplifies the evolving definition of “platinum resistance” in the post-PARPi era. Traditionally, PROC is defined by a platinum-free interval (PFI) of less than 6 months. In this case, although the nominal PFI—from the last platinum dose in late November 2023 to biochemical progression on maintenance olaparib in July 2024—was approximately 8 months, suggesting platinum sensitivity under conventional criteria (>6 months), early signs of resistance were apparent during the postoperative adjuvant platinum-containing chemotherapy phase, where rising CA-125 and a plateau phase necessitated repeated adjustments and a transition to non-platinum therapy. Furthermore, during the subsequent maintenance therapy lasting about 3 months, tumor markers exhibited slow but continuous elevation, further corroborating this issue. Despite the patient not receiving platinum-based drugs for nearly a year from late November 2023 to November 2024, the post-PARPi context added complexity and uncertainty to potential future platinum rechallenge, indicating an aggressive tumor phenotype that likely bypassed traditional DNA repair pathways and rendered subsequent therapies, such as gemcitabine, ineffective. Additionally, recent clinical studies further substantiate this perspective: failure of PARP inhibitor therapy can modify tumor responses to subsequent treatments, and the conventional definition of “platinum sensitivity” based on PFI has limitations in predicting efficacy. For instance, research demonstrates that PARP inhibitor resistance influences subsequent treatment outcomes and may alter sensitivity to platinum-based drugs (6); in recurrent epithelial ovarian cancer, patients post-PARP inhibitor treatment exhibit uncertainty in their response to subsequent platinum-based chemotherapy (7). Consequently, guidelines from the Gynecologic Cancer Intergroup (GCIG) (8) and the National Comprehensive Cancer Network (NCCN) (9) highlight the limitations of relying solely on PFI to define platinum sensitivity and emphasize the need for flexibility in clinical decision-making. Overall, these insights underscore the urgent necessity to transition from PFI-based sequential treatment paradigms to biomarker-driven strategies in the post-PARPi setting.

To contextualize the significance of the response to YL205, we review the evolving therapeutic landscape of PROC, contrasting historical standards with the emerging paradigm of ADCs. Historically, PROC management has relied on non-platinum chemotherapies (e.g., paclitaxel, pegylated liposomal doxorubicin, topotecan, gemcitabine), yielding objective response rates (ORRs) of 10–15% and median progression-free survival (PFS) of ~3.5 months. Recent trials reveal variability; for instance, weekly paclitaxel achieves ORRs of ~30% and PFS of 5.5 months (5), yet adding bevacizumab (as in the AURELIA trial) provides no overall survival benefit (10). This underscores the limitations of conventional approaches and sets the stage for innovative ADC-based strategies.

### 4.2 ADC landscape in PROC

ADCs have emerged as transformative agents in PROC by delivering cytotoxic payloads to antigen-expressing tumors (11, 12). The approval of mirvetuximab soravtansine (MIRV) in 2023 and confirmatory MIRASOL phase III data marked the first ADC triumph in PROC (13). Trastuzumab deruxtecan (T-DXd), a HER2-targeting ADC, showed an ORR of 57.5% in HER2-expressing ovarian cancer in the DESTINY-PanTumor02 trial (14). NaPi2b, overexpressed in ovarian cancers, is a promising target (15). Early studies with lifastuzumab vedotin showed higher ORR than doxorubicin (34% vs. 15%), though PFS improvement was nonsignificant (16). YL205 represents a next-generation NaPi2b ADC, with this case demonstrating its potential for complete responses in biomarker-selected patients. Other NaPi2b ADCs, such as TUB-040, have shown robust activity in phase I/IIa trials for PROC, with ORRs exceeding 50%, a DCR of 96%, and complete responses across dose levels in heavily pretreated patients (17, 18). Key trials confirm the superiority of next-gen ADCs, with ORRs up to 59% for optimized NaPi2b-targeting agents (19, 20). The CDH6-targeted raludotatug deruxtecan (R-DXd) demonstrated an ORR of 50.5% in the REJOICE-Ovarian01 trial (21). The EGFR×HER3 bispecific Iza-bren (BL-B01D1) achieved a confirmed objective response rate (cORR) of 49.0% and median PFS of 7.0 months in PROC subgroups (22, 23). Overall, the literature underscores a shift toward biomarker-driven ADC therapies, with repeated profiling essential for trial eligibility.

**Table 1** provides a comparative overview of representative ADCs. Compared with first-generation NaPi2b ADCs using MMAE payloads, YL205 employs a different linker-payload design that may help circumvent taxane-related resistance mechanisms.

**Table 1:** Comparative profile of representative antibody-drug conjugates (ADCs) for platinum-resistant ovarian cancer (PROC).

Target/agent	Payload (DAR)	Key trial/population	ORR (confirmed)	mPFS/mDOR	Notable toxicities
FRαMirvetuximab soravtansine (MIRV)	DM4	MIRASOL Phase 3(FRα-high PROC, 1–3 prior lines)	41.9–42.3%	mPFS: 5.59–5.62 momDOR: ~6.9 mo	Ocular (keratopathy, blurred vision; Gr≥3 ~16%), GI toxicity
HER2Trastuzumab deruxtecan (T-DXd)	DXd (Topo I)	DESTINY-PanTumor02(HER2-expressing ovarian cohort)	~45% (overall ovarian)~63.6% (IHC 3+)	mPFS: 5.9–6.9 mo (overall ovarian)11.9 mo (IHC 3+)	ILD/pneumonitis (~10–12%), hematologic toxicity
CDH6Raludotatug deruxtecan (R-DXd)	DXd (Topo I)	REJOICE-Ovarian01 Phase 2(PROC)	50.5% (across doses)50.0% (5.6 mg/kg)	Early data: mDOR ~8–11 mo	GI toxicity, fatigue, hematologic toxicity
NaPi2b (1st gen)Lifastuzumab vedotin	MMAE	Phase 2 (vs PLD)(PROC, high NaPi2b)	34–36% (high NaPi2b)	mPFS ~5.3 mo	Neuropathy, hematologic toxicity (taxane-like)
NaPi2b (next-gen)TUB-040	Exatecan (Topo I)	NAPISTAR 1-01 Phase 1/2a(PROC)	Unconfirmed: 49–59%Confirmed: ~33–50%	Responses ongoing (early data)	Manageable hematologic toxicity; minimal ocular/ILD events
NaPi2b (next-gen)YL205 (this report)	YL0010014 (Topo I, DAR 8)	Phase I/II (YL205-CN-101-01)(NaPi2b-high [83% TPS], heavily pretreated post-PARPi/USP1i)	CR (this case)	CR achieved; as of Apr 2026, sustained ongoing CR for >48 weeks[this case]	Isolated Grade 2 leukopenia (Nadir 1.1 × 10^9^/L); No ocular toxicity, anemia, or thrombocytopenia observed.

ADC, antibody−drug conjugate; PROC, platinum−resistant ovarian cancer; DAR, drug−to−antibody ratio; ORR, objective response rate; CR, complete response; mPFS, median progression−free survival; mDOR, median duration of response; GI, gastrointestinal; ILD, interstitial lung disease; IHC, immunohistochemistry; Topo I, topoisomerase I inhibitor; PLD, pegylated liposomal doxorubicin; PARPi, poly(ADP−ribose) polymerase inhibitor; USP1i, ubiquitin−specific protease 1 inhibitor; TPS, tumor proportion score.

### 4.3 Mechanistic rationale for YL205

**Figure 3:**
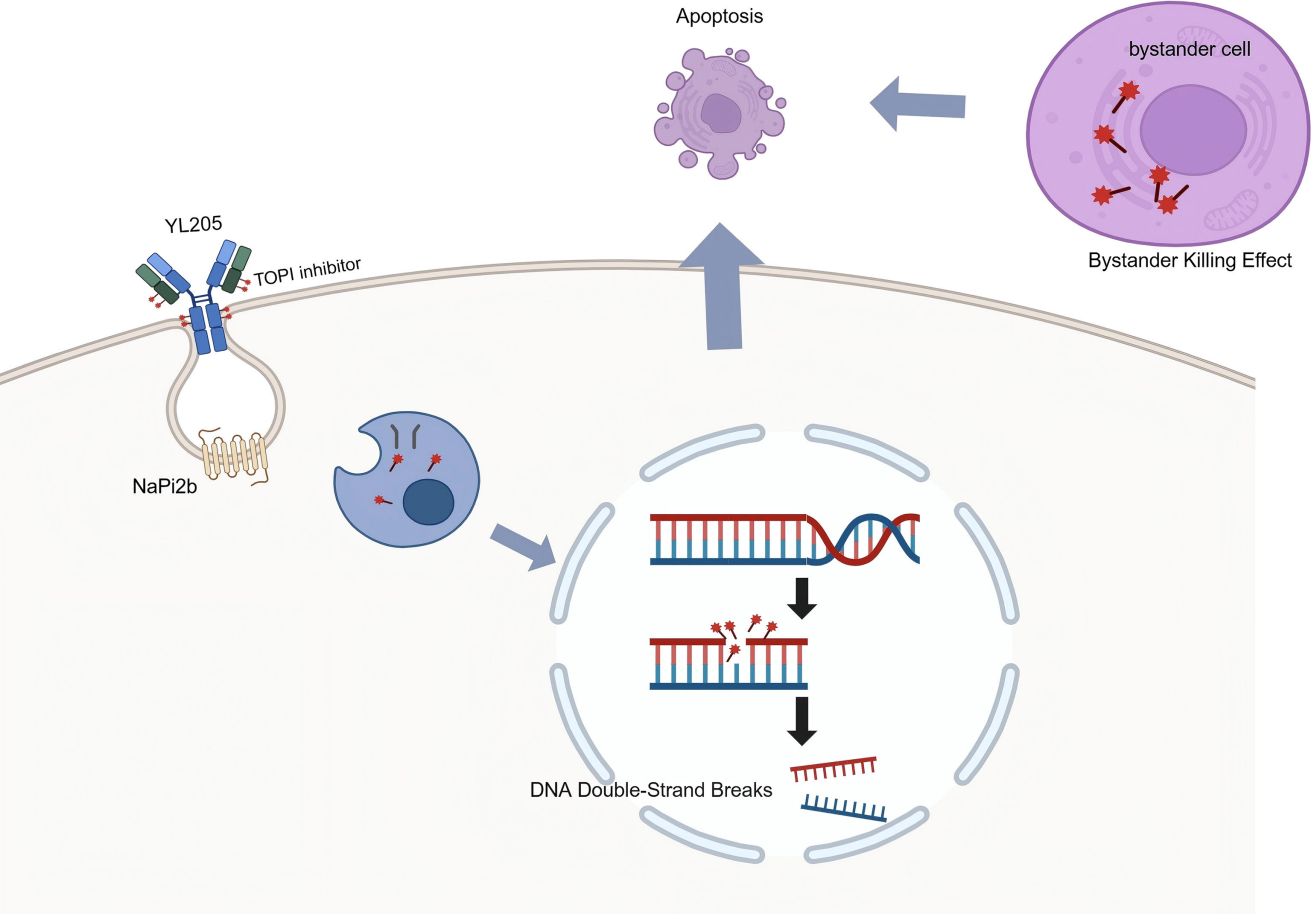
Proposed mechanism of action and bystander effect of YL205. Upon binding to the NaPi2b receptor, the humanized IgG1 antibody facilitates the internalization of YL205 via endocytosis. Following lysosomal degradation, the released TOPI inhibitor (a camptothecin derivative) translocates to the nucleus to induce DNA double-strand breaks and subsequent apoptosis. Additionally, the membrane-permeable payload exerts a “bystander effect” on neighboring antigen-negative tumor cells, addressing the intratumoral heterogeneity characteristic of HGSOC.

While NaPi2b is highly expressed in ovarian cancer (24), first-generation ADCs like lifastuzumab vedotin (MMAE payload) failed due to cross-resistance from prior taxane exposure (4, 16). In contrast, YL205 uses a recombinant humanized IgG1 antibody conjugated to a topoisomerase I inhibitor (YL0010014, camptothecin derivative) with a DAR of 8, inducing DNA damage to bypass taxane-resistance (17). Its efficacy is amplified by the “bystander effect,” where optimized linkers and membrane-permeable payloads eliminate neighboring antigen-negative cells, addressing HGSOC heterogeneity (26) (**Figure 3**). Recent data from TUB-040 validate this design, showing ORRs >50% and CRs in pretreated PROC (18). This parallels our case, emphasizing non-cross-resistant payloads and bystander effects for deep responses. The complete resolution of cervical and retroperitoneal metastases (**Figure 1**) highlights YL205’s tissue penetration, potentially due to favorable pharmacokinetics in lymphatic sites. Thus, YL205’s activity stems from synergistic non-cross-resistant payload and linker design overcoming resistance and heterogeneity (25, 26).

Notably, the profound response to YL205 following USP1 inhibitor (SIM0501) failure suggests a potential therapeutic synergy. USP1 inhibition induces replication fork instability; subsequent exposure to a Topo-I inhibitor payload may exploit this pre-existing replication stress, leading to catastrophic DNA damage. This ‘priming’ effect warrants further investigation as a strategy to enhance ADC efficacy in DNA-repair-deficient tumors.

### 4.4 Safety and management of ADC toxicities

ADCs’ unique safety profiles necessitate vigilant oversight, with toxicities often payload-driven (e.g., neuropathy from microtubule inhibitors, ocular events from DM4 in MIRV) (28). For topoisomerase I-based ADCs like T-DXd, ILD/pneumonitis risks require pulmonary monitoring (14); R-DXd shows gastrointestinal toxicity and fatigue (21); BL-B01D1 hematologic effects need blood count surveillance (22). YL205 reported manageable isolated Grade 2 leukopenia, consistent with class effects, resolved via G-CSF (27).

Despite the zero-event profile observed in our patient, proactive ocular toxicity management remains essential to broadly optimize the therapeutic index of ADCs. We recommend a comprehensive protocol that integrates baseline ophthalmic assessments with routine pre-dose screening for xerophthalmia and blurred vision. Prophylactic care should prioritize the consistent use of preservative-free artificial tears and lifestyle modifications, including the avoidance of contact lenses and the use of sunglasses to mitigate photophobia (28, 29). Furthermore, nursing-led early warning systems are critical to facilitating rapid multidisciplinary intervention—ranging from specialist referral for corticosteroid therapy to potential dose modifications—thereby ensuring treatment adherence and preventing irreversible visual decline (30).

Systematic reviews emphasize early intervention for tolerability (31–33). Successful ADC integration in PROC therapy thus relies on a multidisciplinary management approach to optimize the therapeutic index while preserving patient quality of life.

### 4.5 Predictive biomarkers and future perspectives

This case supports the utility of NaPi2b IHC for patient selection. Future development should incorporate repeated molecular profiling and explore rational combinations with DNA damage response inhibitors or immunotherapies. Larger trials are required to determine optimal sequencing, durability of response, and comparative effectiveness of NaPi2b-directed ADCs in PROC.

### 4.6 Clinical implications

In contemporary PROC management, biomarker-driven strategies augment traditional chemotherapy. Routine profiling for targets: prioritize MIRV for FRα-high, R-DXd for CDH6, Iza-bren for EGFR/HER3. Reassess at progression, tailoring to biomarkers, toxicities, and preferences. As in this case, persistent profiling enables access to YL205, yielding profound responses post-multiple lines. Consensus guidelines emphasize trial participation and flexible platinum resistance definitions amid evolving ADC options.

#### 4.6.1 Strengths and limitations

Strengths include detailed longitudinal follow-up, complication management, and biomarker-driven approach, demonstrating YL205’s efficacy in real-world settings. Limitations: single-case generalizability issues; intermediate follow-up; no controls for causality. Larger trials needed.

#### 4.6.2 Take-away lessons

Emphasize repeated molecular profiling, next-generation ADCs for resistance, post-surgical complication management, and proactive toxicity handling for deep PROC responses.

## 5 Conclusions

This case and accompanying review underscore the formidable challenges inherent in managing aggressive, multi-resistant HGSOC and illuminate the transformative potential of next-generation ADCs, exemplified by the exceptional complete response to YL205. Achieving deep, sustained clinical outcomes in the increasingly complex post-PARPi era necessitates a clinical paradigm shift toward comprehensive, longitudinal molecular profiling to identify actionable targets such as NaPi2b. While ADCs provide a potent addition to the therapeutic armamentarium in PROC, their clinical success is predicated on the vigilant, multidisciplinary management of unique toxicity profiles to ensure patient safety and treatment adherence. Ultimately, future randomized controlled trials are imperative to define the optimal sequencing, long-term durability, and comparative safety of these agents within the rapidly evolving landscape of platinum-resistant ovarian cancer.

## Data Availability

The original contributions presented in the study are included in the article/supplementary material. Further inquiries can be directed to the corresponding author.
